# Targeted Delivery of Arctigenin Using Sialic Acid Conjugate-Modified Liposomes for the Treatment of Breast Cancer

**DOI:** 10.3390/molecules29010278

**Published:** 2024-01-04

**Authors:** Shunfang Liu, Yaozhen He, Minding Feng, Yongtong Huang, Wenhao Wu, Jiu Wang

**Affiliations:** 1 Guangdong Provincial Key Laboratory of Advanced Drug Delivery Systems, Center for New Drug Research and Development, Guangdong Pharmaceutical University, Guangzhou 510006, China; wendy07131998@163.com (S.L.); heyaozhen1998@163.com (Y.H.); minding0717@163.com (M.F.); hyt1292400877@163.com (Y.H.);; 2 Guangdong High Education Institutes Engineering Research Center of Modified-Released Pharmaceutical Products, School of Pharmacy, Guangdong Pharmaceutical University, Guangzhou 510006, China; 3 Guangdong Provincial Engineering Center of Topical Precise Drug Delivery System, School of Traditional Chinese Medicine, Guangdong Pharmaceutical University, Guangzhou 510006, China

**Keywords:** sialic acid receptor, nanoliposomes, targeted delivery, arctigenin, antitumor in vivo

## Abstract

Arctigenin (ATG) is a broad-spectrum antitumor drug with an excellent inhibitory effect on malignant tumors such as breast cancer, glioblastoma, liver cancer, and colon cancer. However, the clinical application of ATG is limited by its poor water solubility and quick hydrolysis in the liver, intestine, and plasma, which might hinder its application. Sialic acid (SA) recognizes selectin receptors overexpressed on the surface of tumor-associated macrophages. In this study, SA was conjugated with octadecylamine (ODA) to prepare SA-ODA, which was employed to prepare SA functionalized nanoliposomes (SA-Lip) to achieve breast cancer targeting. The formulations were finely optimized using the Box–Behnken design to achieve higher ATG loading. The size, ζ potential, entrapment efficiency, drug loading, and release behavior of ATG@SA-Lip were fully investigated in comparison with conventional ATG@Lip. The ATG@SA-Lip displayed more potent cytotoxicity and higher cellular internalization compared to ATG@Sol and ATG@Lip in both MCF7 and 4T1 cells. Notably, ATG@SA-Lip showed the lowest impact on the immune system. Our study demonstrates that SA-Lip has strong potential as a delivery system for the targeted delivery of ATG.

## 1. Introduction

**Figure 1:**
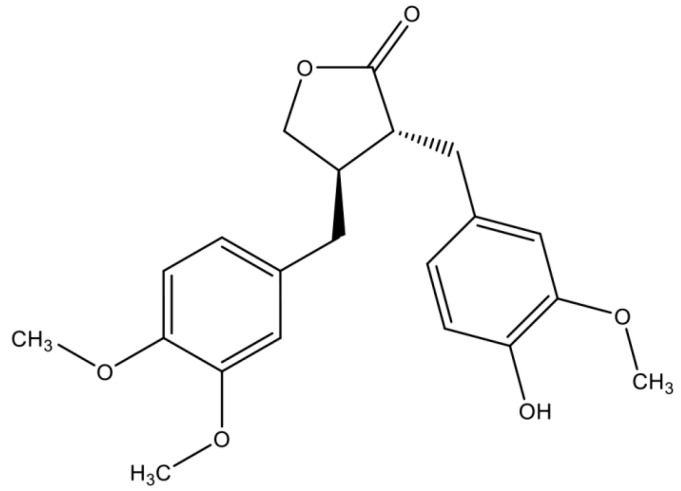
Chemical structure of arctigenin.

Arctigenin (ATG) (Figure 1) is a phenylpropanoid dibenzyl butyrolactone lignan compound, one of the primary bioactive ingredients from the medicinal plant *Arctium lappa* L. ATG possesses notable pharmacological activity, such as anti-inflammatory [1,2], immunoregulatory, antiviral, PP2A activation [3], and anticancer activities [4,5]. Many studies in the literature show that ATG is a broad-spectrum antitumor drug with an excellent inhibitory effect on malignant tumors such as breast cancer [6,7], glioblastoma [8], liver cancer [9,10], and colon cancer [11,12,13]. The molecular mechanisms behind ATG’s biological effects have been intensively studied [4,13,14]. The anticancer mechanisms include cytotoxicity [15], suppression of proliferation [16], induction of apoptosis [17], repression of angiogenesis, migration and invasion of cancer cells [7], enhancement of drug cytotoxicity [18], and immunomodulatory effects [19]. ATG regulates the immune system as an immunomodulator through its anti-inflammatory effect, thus exerting an antitumor effect. It is speculated that M2 macrophages are immune cell targets for ATG in breast cancer treatment. However, the clinical application of ATG is limited by many obstacles, such as poor water solubility, extensive glucuronidation, and hydrolysis in many tissues, such as in the liver, intestine, and plasma.

Liposomes are spherical vesicles composed of a bilayer and/or a concentric series of multiple bilayers enclosing an aqueous medium conformed by amphipathic molecules such as phospholipids and cholesterol [20,21,22]. This unique lipid bilayer and hydrophilic cavity structure can carry and deliver hydrophobic and hydrophilic molecules simultaneously. The amphiphilic characteristics of phospholipids are similar to those of biomembranes, allowing for good interaction between liposomes and biomembranes and promoting cellular uptake. Liposomes can be functionalized by adding special components to achieve more apparent advantages, such as prolonged systemic circulation and reduced systemic and off-target toxicity. Novel liposomes with specific biological effects can be produced by modifying the structure and surface of lipid molecules [23,24].

Sialic acid (SA) is a derivative of 9-carbon monosaccharides, typically present as terminal sugars on cell surface glycoproteins or glycolipids [25,26,27]. Tumor cells evade phagocytosis through a high expression of SA-modified glycans [28,29]. In recent years, binding proteins of SA have been discovered, among which sialic acid-binding immunoglobulin-like lectins (Siglecs), also known as sialic acid adhesins, play an important role in the pro-inflammatory response of macrophages. Some studies have shown that Siglecs are highly expressed on the surface of tumor-related macrophages in mammals [30,31]. Most Siglecs are endocytic receptors, making it possible to deliver cytotoxic drugs or immune modulators to target cells by targeting Siglecs [25,31,32,33]. These findings suggest that modifying sialic acid on carriers to increase the targeting effect of the formulation is a promising strategy for cancer treatment. It plays a key role in the stealth and targeting ligand. Recently, Kim et al. used SA-coated Au-NPs to target cancer and effectively evade the immune system [34]. She et al. [35] prepared pixantrone-loaded liposomes modified with SA-octadecyl amine conjugates, which displayed highly effective anticancer and life-prolonging effects. Zheng et al. [36] reported that the cellular uptake of SA-modified selenium nanoparticles (SA-Se-NPs) in HeLa cells was three times that of Se-NPs. These studies indicate that SA parts could be used to strengthen the cellular uptake and cytotoxicity of modified supporters.

In the present study, the sialic acid–octadecylamine (SA-ODA) complex was synthesized via an amidation reaction and used to modify the surface of ATG-containing nanoliposomes (ATG@Lip). The formulation and process parameters were systematically studied and optimized using the Box–Behnken design to achieve higher ATG loading. The size, ζ potential, entrapment efficiency (EE), drug loading (DL) capacity, and release behavior of ATG@SA-Lip were fully investigated in comparison with conventional ATG@Lip. The enhanced cytotoxicity, cellular uptake, and in vivo antitumor activity of ATG@SA-Lip were examined to verify the tumor-targeting ability of decorated SA.

## 2. Results and Discussion

### 2.1. Preparation and Formulation Optimization of ATG@Lip

#### 2.1.1. Preparation Method

Two methods, the film dispersion method (FDM) and the ethanol injection method (EIM), were chosen to explore the preparation of ATG@Lip. The results showed that ATG@Lip prepared by the two methods had a transparent light blue milky appearance. ATG@Lip prepared with EIM had smaller PDI, larger particle size, and poorer stability than that prepared with FDM. ATG@Lip prepared with FDM was stable for 14 days, while that prepared with EIM was stable for only 3 days. Therefore, the thin film dispersion method was used for subsequent experiments.

#### 2.1.2. Optimization of Prescription by Single-Factor Experiments

**Figure 2:**
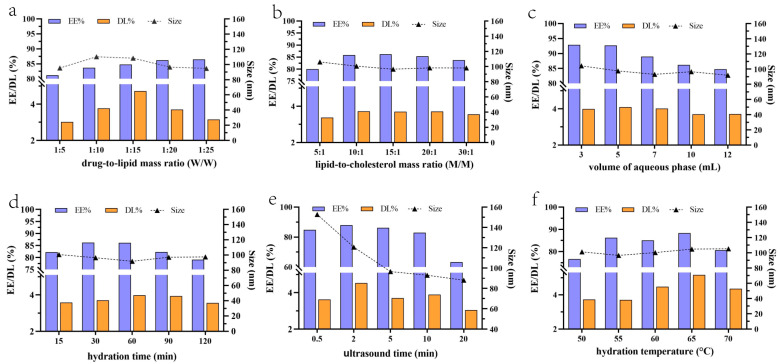
Effect of formulation and process parameters on EE, DL, and Size: (**a**) drug-to-lipid mass ratio; (**b**) lipid-to-cholesterol mass ratio; (**c**) volume of aqueous phase; (**d**) hydration time; (**e**) ultrasound time; (**f**) hydration temperature.

Size, EE, and DL are the critical quality parameters of nanoliposomes. In single-factor experiments, we examined the effects of the formulation and almost all process parameters on size, EE, and DL. As shown in Figure 2a, the particle size decreased slightly with a decreased drug-to-lipid mass ratio (increased lipid mass). The EE continuously increased until reaching a plateau with a decreasing drug-to-lipid mass ratio. DL showed a trend of first decreasing and then increasing with the drug-to-lipid mass ratio and had the maximum value when the ratio was 1:15. The formulations precipitated solid drugs when the drug-to-lipid mass ratio was 1:5 and 1:10, indicating that drug overloading would result in unstable liposomes. According to the entropy weight method, the comprehensive score of the five formulations was 25.72, 46.40, 76.41, 49.09, and 33.01. These scores indicate that the drug-to-lipid mass ratio is an essential factor.

Cholesterol can regulate the fluidity of phospholipid bilayers to reduce membrane permeability and drug leakage. It not only allows the lipid membrane to maintain flexibility and enhances the ability of lipid vesicles to resist changes in external conditions, but also protects phospholipids against oxidation. The amount of cholesterol has an appropriate range; an amount beyond this range, over the membrane load, will lead to liposome rupture, which will adversely affect size, EE, and DL [37]. In our study, the particle size was significantly unaffected by the mass ratio of S75 to cholesterol, as shown in Figure 2b. When the mass ratio was between 10:1 and 20:1, there was no effect on EE, and when it was out of the range (below or above), it had an adverse effect on EE and DL. This might result from interactions between the phospholipid’s oxygen atoms and the cholesterol’s hydroxyl group. This binding reduces the fluidity of the phospholipid bilayer and enhances the rigidity and density of the membrane. The appropriate membrane strength is conducive to the stability of liposomes and the reduction in drug leakage due to excessive membrane fluidity. However, excessive cholesterol will reduce the space of drug loading. The comprehensive score of the five formulations calculated with the entropy weight method was 32.96, 48.89, 49.09, 48.87, and 43.13. The amount of cholesterol had little effect on the three indicators in this range. The mass ratio of S75 to cholesterol of 15:1 was chosen for the subsequent study.

The volume of the hydration medium has a significant influence on the preparation of liposomes. If the volume is too small, the lipid concentration will be too high and the viscosity will be large; if the volume is too large, the particle size of liposomes may be larger or the lipid concentration will be so low that liposomes will be unable to form.

The particle size showed little change (from 92 to 105 nm) with increased aqueous phase volume from 3 to 12 mL (Figure 2c). EE and DL decreased with increased aqueous phase volume and had their maximum value at 3 mL. However, it was difficult to hydrate, and the hydration liquid was too sticky when the volume was 3 mL, which affected the preparation process. The above phenomena improved significantly when the volume was increased to 5 mL even though the hydration fluid remained slightly sticky. The comprehensive score of the five formulations was calculated to be 60.31, 64.83, 61.49, 49.09, and 49.79 based on the entropy weight method. Considering the dose, we selected a hydration medium volume of 5–10 mL in the subsequent study.

The effects of process parameters (hydration time, ultrasound time, and hydration temperature) on size, EE, and PDI are summarized in Figure 2d–f with the same formulations. EE and DL showed a trend of first increasing and then decreasing gradually with hydration time increasing from 15 to 120 min, just the opposite of size (Figure 2d). All parameters had a maximum value of 60 min. This shows that appropriately increasing the hydration time facilitates the encapsulation of the drug in the liposome. Still, after 60 min, the drug leaked as the hydration time increased, resulting in reduced EE and DL. Calculating by the entropy weight method, the comprehensive score of the five formulations was 44.42, 49.09, 58.95, 56.07, and 39.65. A hydration time of 60, with the highest comprehensive score, was chosen for the subsequent study.

Ultrasound time had a noticeable effect on particle size. As shown in Figure 2e, the size decreased sharply when ultrasound time increased from 0.5 to 5 min, then slowly decreased from 5 to 20 min. In contrast, excessive ultrasound time has an adverse effect on drug loading and EE. Drug loading and EE decreased with increasing ultrasound time, which might result from the rupture of liposome members. Our test results are in good agreement with many previous studies. The comprehensive score of the five formulations was 31.55, 69.36, 49.09, 53.74, and 17.14, indicating that ultrasound time is an essential factor in liposome preparation. An ultrasound time of 2–10 min was chosen for optimization in the subsequent study.

The phase transition temperature (T_c_) of phospholipids is the temperature at which the phospholipids transition from gel state (highly ordered) to liquid crystalline state (disordered) [38]. Liposome membrane permeability increases with increasing acyl chain activity at the phase transition temperature. T_c_ is the critical parameter of liposome preparation and generally requires that the temperature be higher than T_c_. If the preparation temperature is lower than T_c_, the obtained rigid liposomes will have a hard time deforming and changing particle size. The obtained liposomes would become flexible and could be deformed and change particle size by extruding only above T_c_. As shown in Figure 2f, EE and DL increased with hydration temperature rising from 50 to 65 °C; however, they decreased when hydration temperature reached 70 °C, and EE was less than 80%. The particle size changed from 97 to 105 nm with increasing hydration temperature, but this was not affected significantly. The comprehensive score of the five formulations was 42.90, 49.09, 71.03, 92.83, and 63.65. The hydration temperature was 65 °C, with the highest comprehensive score. However, the higher the preparation temperature, the greater the risk of phospholipid oxidation, leading to formulation instability. So, the hydration temperature of 60 °C was chosen for subsequent study.

The values of H’_j_ and W’_j_ for EE, DL, and particle size (Size) after process optimization are listed in Table S1 (Supplementary Materials).

#### 2.1.3. Optimization of Prescription by Box–Behnken Design

Three main influencing factors were determined based on the single factor results. The scope and level of influencing factors are listed in Table S2 (Supplementary Materials). All experiments were designed using Design-Expert 13 software. Seventeen trials were randomly arranged and performed. The drug-to-lipid mass ratio (X_1_, M1/M2), the volume of the aqueous phase (X_2_), and ultrasound time (X_3_) were independent variables, and EE (Y1), DL (Y2), and size (Y3) were the dependent variables. The weight coefficients of the three dependent variables were given by EWM. The weights of size, EE, and DL were 0.3515, 0.3617, and 0.2867, respectively. Then, the comprehensive score was calculated. The three high, medium, and low levels of the three factors were 1, 0, and −1, respectively. Box–Behnken design and variance analysis results are given in Tables S3 and S4 (Supplementary Materials). The regression equation for the effects of various factors on the comprehensive score is:

Y = 59.96 − 11.77X_1_ − 17.02X_2_ + 2.68X_3_ − 0.3592X_1_X_2_ − 3.53X_1_X_3_ + 1.97X_2_X_3_ − 7.16X_1_^2^ − 1.29X_2_^2^ − 11.59X_3_^2^

**Figure 3:**
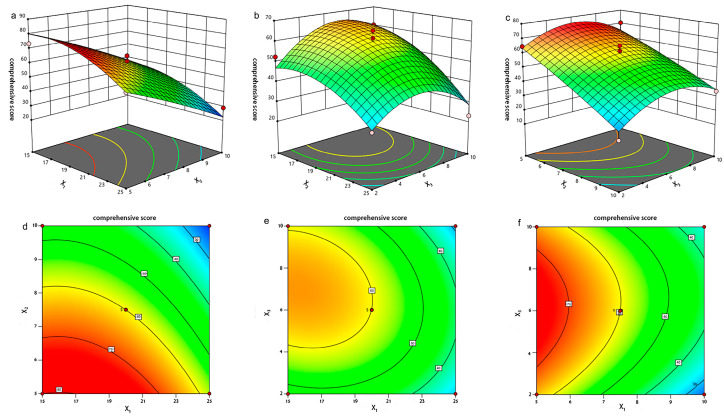
Response surface plots for effects of drug-to-lipid mass ratio (X_1_), volume of aqueous phase (X_2_), and ultrasound time (X_3_) on the score: (**a**,**d**) interaction between X_1_ and X_2_; (**b**,**e**) interaction between X_1_ and X_3_; (**c**,**f**) interaction between X_2_ and X_3_.

As shown in Table S4 (Supplementary Materials), the significance (*p*) of the model obtained by BBD was 0.0014 < 0.01, indicating that the model was significant. The regression coefficient *R*^2^ was 0.9430, indicating that the regression model could interpret experimental data with high reliability. The influence of each factor on the comprehensive score was X_2_ > X_1_ > X_3_ (X_2_ and X_1_ were significant, *p* < 0.01; X_3_ was not significant, *p* > 0.05). Combining the results of ultrasound time in the single factor experiment, we speculated that an ultrasound time of 2 to 10 min mainly affected particle size (Y3) but did not significantly affect EE (Y1) and DL (Y2). The interaction of X_1_X_2_, X_1_X_3_, and X_2_X_3_ in the interaction term did not influence the comprehensiveness. Analyze and draw 3D response surface plots of each factor on the comprehensive score using Design-Expert 13 software. The steeper the curve in the plots, the greater the influence of the factor. As shown in Figure 3, the surface of factors X_1_ and X_3_ was the steepest, followed by X_2_ and X_3_. The surface of factors X_1_ and X_2_ was the smoothest. This is consistent with the results of the regression equation model’s analysis of variance.

Through the optimization and prediction of prescription factor levels with Design-Expert 13, the optimal nanoliposome preparation process was obtained as a drug-to-lipid mass ratio of 1:15.83, volume of aqueous medium of 5 mL, ultrasound time of 6.63 min, and predicted comprehensive score of 80.51.

Considering the feasibility of the experimental operation, the process was modified to a drug-to-lipid mass ratio of 1:15.8, volume of aqueous medium of 5 mL, and ultrasound time of 6.6 min. Three batches of ATG nanoliposomes were prepared under optimal conditions to verify the validity of the optimization formulation and technology. EE% (90.31 ± 0.49), DL% (4.80 ± 0.04), and size (97.4 ± 2.1 nm) were detected, and the comprehensive score was 79.86 ± 1.09, which was close to the predicted value of 80.51. This proves that EWM combined with BBD could be utilized to optimize the nanoliposome preparation process.

### 2.2. SA-ODA Toxicity Assay and ATG@SA-Lip Preparation

The toxicity of the formulation (SA-Lip) to 4T1 cells increased with the amount of SA-ODA. When the amount of SA-ODA did not exceed 3 mg/mL, the cell viability exceeded 95%, and when the amount reached 4 mg/mL, the cell survival rate was less than 80%. Therefore, it was determined that in subsequent experiments, the amount of SA-ODA in SA-modified liposomes should not exceed 3 mg/mL. Based on the above data, the formulation of ATG nanoliposomes was determined as follows: 21 mg/mL ofCS-95, 0.1mg/mL of ODA (ATG@Lip), and 0.04 mg/mL of ODA and 0.06 mg/mL of SA-ODA (ATG@SA-Lip), drug-to-lipid ratio of 1:15.8, lipid-to-cholesterol ratio of 15, hydration time of 60 min, ultrasound time of 6.6 min, and hydration temperature of 60 °C.

### 2.3. Characterization of Nanoliposomes

**Figure 4:**
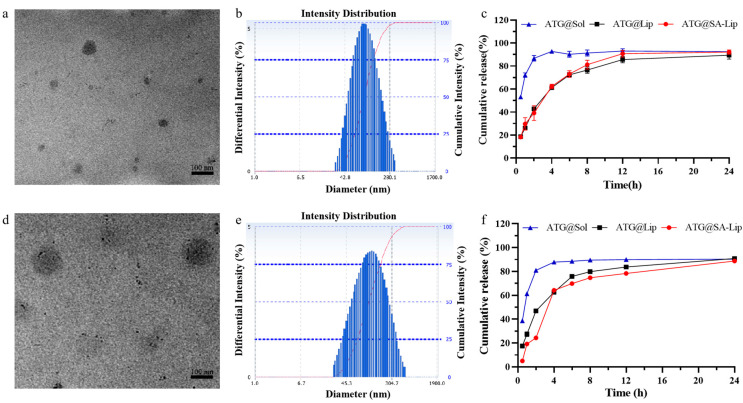
Characterization of (**a**,**b**) ATG@Lip and (**d**,**e**) ATG@SA-Lip. (**a**,**d**) Appearance under TEM (scale bar = 100 nm); (**b**,**e**) particle size by DLS; Cumulative release of ATG formulations at (**c**) pH 5.2 and (**f**) 7.4. Dates are represented as mean ± (SD) (*n* = 3).

The morphology, size, ζ potential, PDI, EE, DL, pH, and drug release of the preparations were investigated. The two formulations were translucent liquid with light blue emulsion. Figure 4a,d presents a representative TEM image. The liposomes exhibited a uniform spherical shape without aggregation. The pH, size (Figure 4b,e), PDI, ζ potential, EE, and DL are shown in Table S5 (Supplementary Materials). The pH of ATG@Lip and ATG@SA-Lip was 6.35 and 6.49, respectively. This is consistent with the requirements of the injector. Except for zeta potential, the size, PDI, EE, and DL of ATG@Lip are similar to those of ATG@SA-lip. Adding ODA-SA will generally decrease zeta potential due to the negative charge of ODA-SA molecules on the surface of the nanoliposome.

In the release experiments, as ATG is insoluble, 0.5% sodium dodecyl sulfate was added to the release medium for the leak condition. Phosphate buffer at pH 7.4 and pH 5.2 represent the normal physiological environment in vivo and the tumor tissue environment, respectively, to study the release behavior of liposomes before and after sialic acid modification. It was examined whether incorporating SA-ODA into the preparations would increase the bilayer permeability and accelerate drug release in the above conditions. The drug release profiles of the ATG formulations were evaluated and are shown in Figure 4c,f. Free ATG released from ATG solution diffused and thoroughly penetrated the dialysis bag at 4 h, while both nanoliposome preparations released ATG sustainably. Moreover, the release profiles of both liposomes were similar under different pH conditions, indicating that the anchoring of SA-ODA did not affect the drug release. To explain the drug release mechanism, we tested four fitting models—zero-order kinetics, first-order kinetics, the Higuchi model, and the Ritger–Peppas model [39]—to fit the released datasets with Origin software 8 Pro (Table S6, Supplementary Materials). Among them, the release curves of ATG solution, ATG@Lip, and ATG@SA-Lip had the best fit with the first-order equation. This suggests that the sustained-release process is non-Fickian diffusion. The presented data confirm the tendency previously reported in the literature [40,41,42,43].

### 2.4. Evaluation of Biosafety

The stability of formulations in serum is shown in Table 1. The size of ATG@Lip and ATG@SA-Lip decreased slightly by 9.00% and 8.44%, respectively.

**Figure 5:**
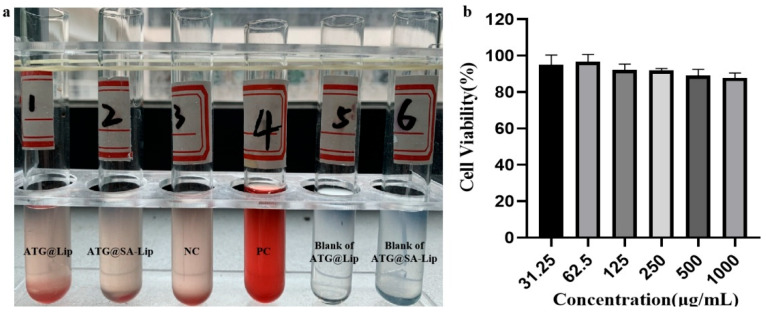
Biocompatibility of nanoliposomes in vitro: (**a**) hemolytic effect of ATG@Lip and ATG@SA-Lip; (**b**) cytotoxicity of SA-Lip.

The blood compatibility of nanoparticles plays a crucial role in their biomedical application, as blood is their main target. When nanoparticles enter the systemic circulation, they lead to REC rupture and hemoglobin release, which can provoke serious adverse reactions. As shown in Figure 5a, the results indicate that the formulations had no hemolytic effects or coagulation within 3 h. This suggests that they did not cause hemolysis after intravenous administration. In the abnormal toxicity test, all mice survived within 48 h after *IV* administration. This indicates that the ATG formulations were biosafe. The in vitro toxicity study by CCK-8 showed that blank SA-liposomes (nanoliposomes with SA aptamers) did not induce potent cytotoxicity (Figure 5b). All the results indicate that the formulations were biosafe.

### 2.5. Cellular Uptake of ATG@Lip and ATG@SA-Lip

**Figure 6:**
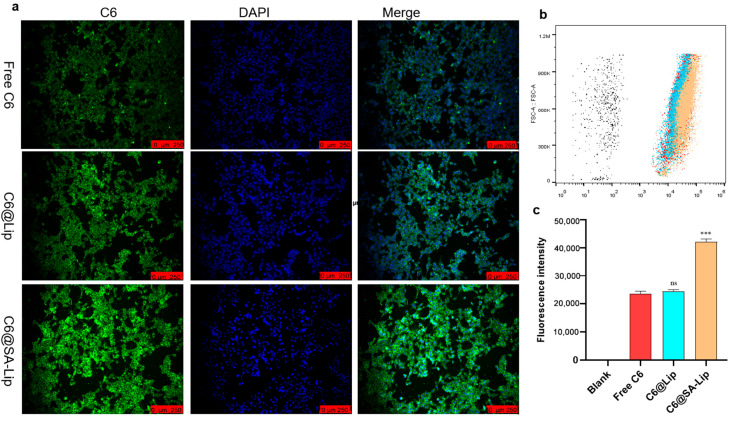
Cellular uptake of fluorescent probes: (**a**) cellular uptake of C-6 labeled formulations on 4T1 cells observed by fluorescence microscopy; (**b**) fluorescence in cell distribution detected by flow cytometry; (**c**) fluorescence intensity calculated with FlowJo Soft 7.6.1 (ns *p* > 0.05, *** *p* < 0.001 compared with free C6).

To investigate the effect of sialic acid on the qualitative uptake of liposomes by 4T1 cells, we employed coumarin-6 as a fluorescence probe for fluorescence microscopy and intensity analysis. As shown in Figure 6a, the qualitative fluorescent images displayed differences in C6 uptake in 4T1 cells treated with different C6 formulations, indicating that C6@SA-Lip had the highest cellular uptake. Moreover, the nanoformulations were located in the cytoplasm around the nucleus. The flow cytometer measured the corresponding quantitative cellular uptake of C6-labeled nanoliposomes in 4T1 cells. As displayed in Figure 6b,c, the mean fluorescence intensity in the C6@SA-Lip group (42,147.67 (±942.99)) was significantly higher compared with the free C6 group (23,707.33 ± 821.20) (*p* < 0.001) and C6@Lip group (24,537.33 (±527.15)) (*p* < 0.001). There was no significant difference in mean fluorescence intensity between the free C6 and C6@Lip groups (*p* > 0.05). This result suggests that nanoliposomes modified with sialic acid promote drug uptake in 4T1 cells.

### 2.6. Cytotoxicity Studies of ATG@Lip and ATG@SA-Lip

**Figure 7:**
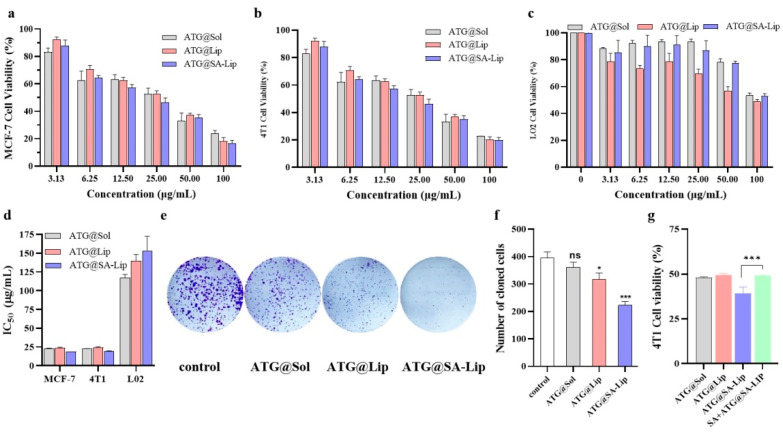
Cytotoxicity of ATG formulations: (**a**–**c**) viability of MCF-7, 4T1and LO2 cells analyzed by CCK-8 assay; (**d**) IC_50_ of ATG formulations for indicated cell lines; (**e**,**f**) colony formation assay of 4T1 cells; (**g**) viability of 4T1 cells treated with formulations of 25 μg/mL ATG (ns *p* > 0.05, * *p* < 0.05, *** *p* < 0.001 compared with control).

The CCK-8 assay and colony formation experiment were used to study the in vitro cytotoxicity of the ATG formulations (Free ATG, ATG@Lip, ATG@SA-Lip) in MCF-7 and 4T1 cells. As displayed in Figure 7a,b, the cell viability ratio of MCF-7 and 4T1 cells treated with various concentrations of ATG formulations gradually decreased with increasing concentration. Half maximal inhibitory concentration (IC_50_) was further calculated. As shown in Figure 7d, the IC_50_ of free ATG was lower than that of ATG-Lip; thus, it is speculated that the nanoliposome (ATG@Lip) sustained the ATG release from its formulation, resulting in weaker cytotoxicity. There are some reports of reduced in vitro cytotoxicity after liposome wrapping of drugs [35,44]. Notably, ATG@SA-Lip (IC_50_ = 18.77 and 19.31 μg/mL to MCF-7 and 4T1, respectively) had the most potent cytotoxicity compared to ATG@Lip (IC_50_ = 23.92 and 24.38 μg/mL) and ATG solution (IC_50_ = 22.68 and 22.37 μg/mL). This phenomenon can be attributed to the strong affinity of SA-functionalized nanoliposomes to BC cells by the overexpressed Siglec, leading to enhanced nanoliposome uptake through receptor-mediated endocytosis [30,33,34,45]. This is consistent with the above cellular uptake experiments. We found that the cellular uptake of ATG@Lip and ATG@SA-Lip was significantly higher than that of ATG@sol (Figure 6c), which also indicates that liposomes and modified liposomes could increase drug incorporation. Still, perhaps due to the sustained release, the enhanced cytotoxicity in vitro was not noticeable. Additionally, we studied the cytotoxic activity of ATG@sol, ATG@Lip, and ATC@SA-Lip against normal LO2 cells, as shown in Figure 7c, and we found that ATG@sol, ATG@Lip, and ATC@SA-Lip have low cytotoxic activity toward LO2 cells (IC_50_ > 100 μg/mL).

As shown in Figure 7e,f, the colony formation experiments indicate that ATG@SA-Lip significantly inhibited the clonogenic ability of 4T1 cells compared with ATG solution and ATG@Lip. This is consistent with the results of the cytotoxicity.

The control experiment was performed to validate the mechanism that SA decorated can increase the drug cellular uptake through the siglec’s endocytosis. Cells were pretreated with sialic acid to block siglecs and then treated with Arg@SA-Lip to observe if the cellular toxicity decreased or not. As shown in Figure 7g, the toxicity of ATG@SA-Lip to 4T1 cells pretreated with SA is significantly higher than that of 4T1 cells without SA pretreatment. From the results of the competitive inhibition experiment, it was found that after SA pretreatment of 4T1 cells, the uptake of ATG@SA-Lip by 4T1 cells was significantly reduced compared to that without SA pretreatment. This may be because the pre-supersaturation of sialic acid combined with the siglecs on 4T1 cells resulted in a decreased amount of the SA-modified liposomes entering the cells; hence, the cellular uptake amount was reduced. The phenomenon can be found in other literature [46,47].

### 2.7. Evaluation of Antitumor Activity and Toxicity In Vivo

**Figure 8:**
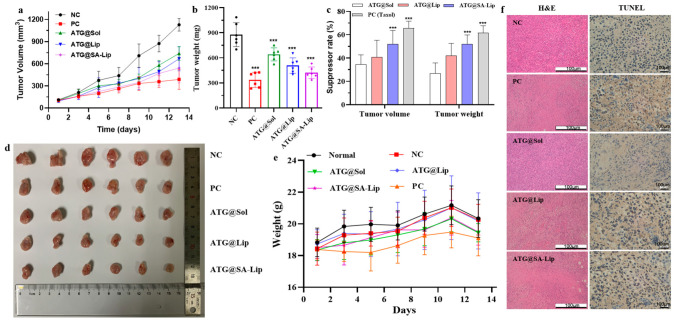
Anticancer activity and toxicity of ATG formulations: (**a**) tumor volume; (**b**) weight of tumors (*** *p* < 0.001); (**c**) tumor suppression rate; (**d**) images of xenograft tumors obtained from mice with different treatments after 14 days; (**e**) body weight of murine model; (**f**) H&E and TUNEL analysis of tumors obtained from sacrificed mice at end of study (*** *p* < 0.001 compared with NC or ATG@Sol).

To study the antitumor effect of ATG formulations on BC in vivo, 4T1 xenografted BALB/c mouse models were generated. As displayed in Figure 8a–d and Table S7 (Supplementary Materials), all ATG formulations significantly reduced the weight and volume of tumors in the mice compared with the NC group (*p* < 0.01). We also found that ATG@SA-Lip efficiently repressed tumor growth compared with ATG@Sol (*p* < 0.01) and ATG@Lip (*p* < 0.05) and had the same tumor suppression effect with PC (*p* > 0.05). The tumor suppression rate was PC > ATG@SA-Lip > ATG@Lip > ATG@Sol. Still, there was no significant difference between the ATG@SA-Lip and PC groups (*p* > 0.05), indicating that the tumor suppression effect of ATG@SA-Lip was close to that of PC. We speculate that this contributes to SA-decorated liposomes, resulting in targeting and EPR.

As shown in Figure 8e, ATG@SA-Lip treatment and positive control did not significantly reduce the body weight of mice compared with the normal group. These findings indicate that ATG@SA-Lip did not cause noticeable side effects. Additionally, the tumor tissue slides were stained with hematoxylin and eosin, and the slides were observed under a microscope to study tumor cell activity. As shown in Figure 8f, there were abundant disordered and densely arranged tumor cells with stained bluish-violet nuclei that maintained their heteromorphism (NC group). There was an extensive pink area (cellular necrosis) in cells from the PC and ATG@SA-Lip groups; in particular, the nuclei almost disappeared in the PC group. The extent of cellular necrosis significantly increased in the following order: ATG@Sol group < ATG@Lip group < ATG@SA-Lip group < PC group.

Furthermore, apoptosis was detected in cancer cells by TUNEL assay. As displayed in Figure 8f, there were statistically significant differences between ATG formulations and NC groups. Apoptotic nuclei were stained dark brown, and a large number of apoptotic tumor cells were found in the PC and ATG@SA-Lip groups, which was far more than the NC and ATG@Sol groups. All data suggest that ATG@SA-Lip induces cell death effects the same as PC.

**Figure 9:**
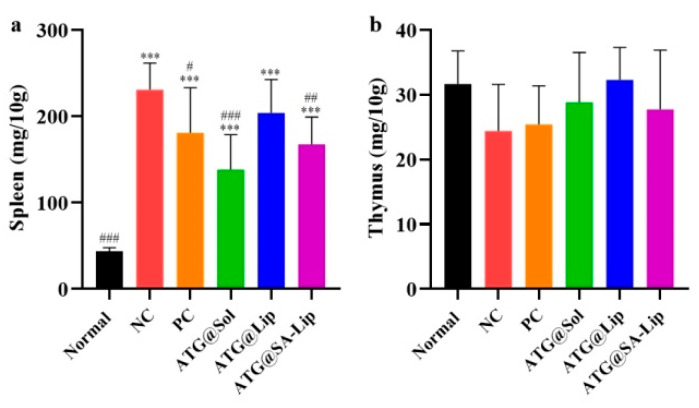
Immune organ index of mice after 13 days of administration: (**a**) spleen index; (**b**) thymus index. (*** *p* < 0.001 compared with normal group, # *p* < 0.05, ## *p* < 0.01, ### *p* < 0.001 compared with NC group).

The spleen and the thymus are the immune organs. As shown in Figure 9, the spleen index of tumor-bearing mice was significantly higher than that of the normal group, indicating that the mice developed an immune response to attack the tumor. Compared to the NC group, the spleen index of all treated groups decreased, indicating that the drug had a therapeutic effect and weakened the body’s immune response. Compared with the normal group, there was no significant difference in the thymus index of the NC group and all treated groups (*p* > 0.05). Compared to the NC group, there was no significant difference in the thymus index of all treatment groups (*p* > 0.05), but there were varying degrees of increase. Moreover, all of the ATG preparation groups had a greater increase in the thymus index than the PC group. It is speculated that the ability of each ATG preparation to restore the body’s immune response to normal was greater than that of the positive drug.

### 2.8. Serum Biochemistry in Mice

On the last day, blood was collected, and all mice were euthanized. Blood cell counting was conducted by automatic hematological analysis. The tumor specimens underwent H&E staining and TUNEL assay. The rates of tumor volume and tumor weight suppression in the PC group and experiment groups were calculated.

Table 2 summarizes the blood cell counting. The results revealed that HGB, RBC, and PLT counts did not differ significantly among the groups. Compared with the normal group, WBC, Mono, Eos, Neu, Lym, and NLR counts increased significantly in the NC group (*p* < 0.05), indicating possible immune action and inflammation in tumor-bearing mice. Compared to the NC group, the above indicators in all experimental groups decreased, indicating that inflammation was alleviated in tumor-bearing mice. Neutrophilia releases various inflammatory factors that promote blood vessel formation and the spread of tumor cells. Compared with the NC group, the number of neutrophils in the PC, ATG@sol, ATG@Lip, and ATG@SA-Lip groups significantly decreased (*p* < 0.05), indicating the occurrence of inhibitory effects on angiogenesis in these experimental groups.

Lymphocytes participate in the body’s immune response, stimulating natural killer cells and macrophages to kill tumor cells directly or release a series of cytokines, thereby activating the immune system. When the number of lymphocytes decreases, the body’s antitumor immune function weakens and cannot effectively kill tumor cells, leading to tumor cell growth and disease progression. Meanwhile, a decreased lymphocyte count induces cell proliferation, promotes tumor development, and increases tissue infiltration by promoting angiogenesis, leading to tumor spread. The number of lymphocytes in the ATG@Lip and ATG@SA-Lip groups was higher than in the positive drug group. This result indicates that the inhibitory effect of arctigenin liposomes on tumor immune escape before and after sialic acid modification was better than that of the positive drug group.

The neutrophil-to-lymphocyte ratio (NLR) indicates systemic inflammation and has been considered a poor prognostic factor in various tumors. An increase in the NLR is associated with a decrease in overall survival and poor prognosis [48]. Compared to the NC group, the NLR of the ATG@SA-Lip group decreased significantly (*p* < 0.05), indicating a better prognosis.

## 3. Materials and Methods

### 3.1. Materials

Soy lecithin (CS-95, SY-SO-220401) and cholesterol (C11884385) were generously provided by AVT Pharmaceutical Tech Co., Ltd. (Shanghai, China). Arctigenin (>98%; MUST-21062310) was purchased from Chengdu Mansite Pharmaceutical Co., Ltd. (Chengdu, China). Sialic acid (SA; H24A9W59567) was purchased from Yuanye Bio-Technology Co., Ltd. (Shanghai, China). N-hydroxysuccinimide (NHS, >98%; C12583367), 1-(3-dimethylaminopropyl)-3-ethylcarbodiimide hydrochloride (EDC·HCl, >98%; C12373846), and octadecylamine (ODA; F2207274) were supplied by Macklin Biochemical Co., Ltd. (Shanghai, China), Coumarin-6 (C6; J1916037) was purchased from Aladdin Reagent (Shanghai, China). 4′,6-Diamidino-2-phenylindole (DAPI; 20220922) was purchased from Solarbio (Beijing, China). Cell Counting Kit-8 (CCK-8; MA0128-4-Oct-19H1) was purchased from Meilun Biotech Co., Ltd. (Dalian, China). DMEM (8122464) was purchased from Gibco Corp. (Carlsbad, CA, USA). Fetal bovine serum (2144324) was purchased from Biological Industries (Kibbutz Beit Haemek, Israel). Other chemicals were of analytical reagent or guaranteed reagent grade and were used without additional purification.

### 3.2. Cell Cultures and Animal Model

Cells: Human liver cells (LO2 cells), mouse 4T1 breast cancer cells (a generous gift from Guangdong Provincial Key Laboratory of Advanced Drug Delivery Systems), and human MCF-7 breast cancer cells (Kunming Cell Bank, Kunming, China) were cultured in DMEM with high glucose (Gibco, Thermo Fisher Scientific, Inc., Waltham, MA, USA). All media were supplemented with 10% FBS (Biological Industries, Kibbutz Beit Haemek, Israel) and penicillin/streptomycin (100 µg/mL), and cells were incubated at 37 °C in a humidified atmosphere with 5% CO_2_.

Animal model: SPF female BALB/c mice (weighing 18–20 g) were provided by Guangdong Medical Experimental Animal Center (Foshan, China).

### 3.3. Preparation and Optimization of Nanoliposome and SA-Functionalized Nanoliposome Formulation

General preparation: Nanoliposomes and SA-functionalized (targeted) nanoliposomes were prepared using the film dispersion method (Bangham). All formulations were prepared using the same process. In brief, appropriate amounts of a lipid mixture of soy lecithin CS-95, cholesterol, octadecylamine (for nanoliposomes) or octadecyl amine-SA (for targeted liposomes) [35,37], and ATG were fully dissolved in dichloromethane and evaporated at 40 °C to form a film, then vacuum-dried for 1 h. The obtained lipid film was hydrated at 60 °C with 2–10 mL purified water for 30 min under high-speed agitation. After hydration, the result was sonicated for 5 min (600 w) using an ultrasonic cell crusher (JY92-II). The suspension was then successively screened through 0.22 μm filter membranes at 25 °C to leach large particles and get ATG@Lip or ATG@SA-Lip.

Single factor trials: To investigate the influence of factors such as the CS-95-to-cholesterol mass ratio (M1/M2), drug-to-lipid mass ratio (M1/M2), and volume of aqueous phase and the process parameters (hydration time, ultrasound time, and hydration temperature) on ATG-Lip preparation, different nanoliposomes were prepared. Entrapment efficiency (EE), drug loading (DL), and particle diameter (Size) were used as evaluation indicators. In this paper, formulation and process optimization were performed by using the entropy weight method (EWM) combined with the single-factor experimental method. The EWM is an objective method that can calculate the weights of various evaluation indicators in a complex system. The objective weight of each target is assigned based on the degree of variation among various targets. For the evaluation indicator, the smaller the information entropy (Hj) and the greater the weight coefficient (Wj), the greater the indicator’s role in the comprehensive evaluation, and vice versa [49,50]. The values of H’j and W’j for the encapsulation ratio, DL, and size were calculated.

Experimental design: The main influencing factors of the formulation and process variables—drug-to-lipid mass ratio (X1), volume of aqueous phase (X2), and sonication time (X3)—based on the preliminary single factor experiment were optimized using response surface methodology (RSM). A Box–Behnken design (BBD) with three factors at three levels was employed to optimize and evaluate the effects of influencing factors on response parameters. The scope and level of each influencing factor were determined based on the results of the single-factor experiment (Table S2, Supplementary Materials). All experiments were designed using Design-Expert 13 software. Seventeen trials were randomly arranged and performed. The results are shown in Table S3 (Supplementary Materials).

### 3.4. Characterization of Nanoliposomes

The appearance of the formulations was observed visually and photographed.

Average size, ζ potential, and polydispersity index (PDI) for ATG@lip and ATG@SA-Lip were measured by dynamic light scattering on a nanoparticle size analyzer (Delsa Nano C, Beckman, Fullerton, CA, USA). Samples were diluted 10–20 times with deionized water. The morphology of nanoliposomes was observed under transmission electron microscopy (JEM2100f, Japan Electronics Corporation, Tokyo, Japan) operated at 5.0 kV accelerating voltage. A drop of the sample was placed on a copper mesh and stained with 2% phosphotungstic acid solution when it was semi-dry. After drying, it was magnified 20,000 times under TEM to observe its morphological characteristics. The entrapment efficiency (EE%) and drug loading rate (DL%) of ATG in nanoliposomes were calculated according to Equations (1) and (2):

(1) $$\;\mathrm{EE}\;\%=\frac{\left(w_{1}-w_{2}\right)}{W_{1}}\times 100\%$$

(2) $$\mathrm{EE}\;\%=\frac{\left(w_{1}-w_{2}\right)}{W_{Total\;lipid}}\times 100\%$$

where W_1_ is the added amount of drug and W_2_ is the amount of free drug. UV-Vis spectra (UV2700, Shimadzu Corporation, Kyoto, Japan) were used to determine the amount of ATG.

Then pH values were determined by a pH meter.

The dialysis bag method was used to examine the in vitro drug release behavior of the formulations. Briefly, 5 mL of ATG suspension or ATG formulation was sealed in the dialysis bag (14,000 DaMw cutoff; Yuanye, China) and dialyzed against 30 mL of PBS (pH 7.4 and 5.2) [51], and 0.5% sodium dodecyl sulfate was added with constant stirring at 37 °C. At different time intervals, release samples were withdrawn and replaced with the same volume of fresh medium. The concentration of ATG in the release medium was analyzed by UV, and the following equation was used to calculate the cumulative release rate (Q_t_). All experiments were conducted in triplicate, and the average results are given.

(3) $$Q_{t}=\frac{30C_{t}+\sum_{t-1}VC_{t-1}}{m}\times 100\%$$

where m represents the total amount of drugs (μg), *C*_t_ is the ATG concentration in the release medium at time t (μg·mL^−1^), *V* is the supplement volume of the release medium (mL), *C*_t−1_ is the concentration of ATG in the release medium at the time point prior to time t (μg·mL^−1^).

### 3.5. Evaluation of Cytotoxicity with CCK-8

The cells were seeded at a density of 4 × 10^3^ cells in 96-well plates and cultured for 24 h. Different ODA-SA or ODA-SA nanoliposome concentrations were incubated with cells for 24 h. Then, 10 μL of CCK-8 reagent was added to each well, followed by incubation for another 2 h at 37 °C. The absorbance (A) of each sample was recorded at 450 nm by a microplate reader (BioTek, Winooski, VT, USA) in 3 independent experiments. Cell viability was calculated according to the following equation:

(4) $$\mathrm{Cell}\;\mathrm{viablity}\;(\%)=(1-\frac{A_{test}-A_{0}}{A_{control}-A_{0}})\times 100\%$$

where A_test_ is the experimental group, A_0_ is the blank group, and A_control_ is the control group.

### 3.6. Evaluation of Biosafety

The cytotoxicity of blank preparations (SA-Lip), in vitro stability and hemolytic tests, and in vivo abnormal toxicity of the ATG formulations (ATG@Lip, ATG@SA-Lip) were used to evaluate the safety of the preparations.

Cytotoxicity of blank preparations: The toxicity of different amounts of blank preparation (SA-Lip) toward 4T1 cells was investigated by CCK-8.

Stability studies: Liposomes are often used for intravenous administration. Some factors exist in the blood that can disrupt the liposome structure. It is vital to assess the stability of liposomes in an in vitro setting, simulating the in vivo circulatory system. The ATG formulations obtained after the optimization of various parameters were evaluated for their activity in serum for 48 h. Appropriate samples of formulations were taken, an equal volume of 10% FBS was added, and they were mixed and incubated at 37 °C for 48 h. The samples were withdrawn and evaluated for changes in particle size at 0, 3, 6, 12, 24, and 48 h.

Hemolysis: The ability of the ATG formulations to cause acute hemolysis in vitro was evaluated by hemolysis and coagulation tests based on the Chinese Pharmacopoeia (2020 edition, general rule in the fourth book) and [52].

Abnormal toxicity: Abnormal toxicity in vivo was evaluated with a single intravenous administration of ATG formulations. Briefly, 10 mice (Kunming) weighing about 20 g were randomized into two groups, and 0.2 mL ATG@Lip or ATG@SA-Lip was injected into the tail vein. The mice had access to water and food and were observed to determine whether they had abnormal manifestations over 48 h.

### 3.7. Colony-Forming Experiment

For this experiment, 4T1 cells were seeded into 6-well plates at a density of 500 cells/well. After adhering to the walls, the cells were treated with 1 mL of different ATG formulations and incubated for 24 h, then 2 mL DMEM was added, followed by culture with 5% CO_2_ at 37 °C. After culturing for 10 days, the cells were washed and fixed with paraformaldehyde and then stained with 0.2% gentian violet. The cloning results were observed and photographed under a light microscope (Olympus CKX53, Olympus Corporation, Tokyo, Japan). Finally, the number of clones in the plate was counted using ImageJ software Primer, and the clone formation rate was calculated.

The cloning efficiency was calculated by Formula (5):

Clone formation rate % = N_c_/N_s_ × 100 (5)

where N_c_ is the number of clones, and N_s_ is the number of seeded cells.

### 3.8. Cellular Uptake of ATG@Lip and ATG@SA-Lip

The cellular uptake of coumarin-6 (C6) labeled formulations was evaluated in 4T1 cells. C6 was added to the lipid matrix instead of ATG to mark nanoliposomes. Then, C6-labeled nanoliposomes were prepared using the film dispersion method as described previously. Finally, C6-labeled liposomes containing C6 (1 mg/mL) were obtained. Cells were planted in 6-well plates at a density of 1 × 10^5^ cells/well and cultured for 24 h; then, the cells were treated with C6-Lip or C6@SA-Lip for 2 h. To label the nucleus, DAPI solution (5 µg/mL) was added to the wells and incubated for 15 min. The cells were fixed with 4% polyformaldehyde solution for 20 min and observed under an inverted fluorescence microscope (DMi8, Leica, Wetzler, Germany). The cellular uptake efficiency was further quantified by flow cytometry (Attune, Thermo Fisher Scientific, Waltham, MA, USA). The cells were treated as described previously. Finally, the cells were resuspended with 1 mL PBS and observed by flow cytometry. The results were further quantified with FlowJo software 7.6.1.

### 3.9. Antitumor Activity and Toxicity Evaluation

The animal experiments were performed based on the Guidelines for Animal Care and Use and were approved by the Institutional Animal Care and Use Committee of Guangdong Pharmaceutical University. Female BALB/c mice (nu/nu) (weighing 18–20 g, 5-week-old) were purchased from Guangdong Medical Experimental Animal Center (Foshan, China) and maintained under SPF conditions with free access to water and a standard diet.

The antitumor efficacy of the ATG formulations was evaluated using a xenograft mouse model with 4T1 cells. Briefly, 4T1 cells were suspended at a density of 5 × 10^6^ cells/mL. Then, 4T1 cells (2.5 × 10^6^) suspended in 100 µL RPMI-1640 medium were injected subcutaneously into the right axillary flank of 30 female BALB/c mice (5 weeks old, weighing 18–20 g). After the tumor reached mung bean size (3 mm diameter, about 7 days), the mice were randomly divided into 5 groups and injections were administered as follows: (I) negative control (NC) group: saline 0.1 mL·10 g^−1^; (II) positive (PC) group: paclitaxel 10 mg·kg^−1^; (III–V) ATG treatment groups: ATG@Sol 20 mg·kg^−1^, ATG@Lip 20 mg·kg^−1^, and ATG@SA-Lip 20 mg·kg^−1^; Normal group (6 mice): saline 0.1 mL·10 g^−1^. The mice were treated 5 times on alternate days. Weight and tumor volume were measured once on alternate days 7 times. On day 13, blood was collected, and then the mice were euthanized. The following hematological parameters were evaluated: hemoglobin concentration (HGB, g/L), erythrocytes (RBC, ×10^12^/L), leukocytes (WBC, ×10^9^/L), platelets (PLT, ×10^9^/L), monocytes (Mono#, ×10^9^/L), basocytes (Bas#, ×10^9^/L), eosinophils (Eos#, ×10^9^/L), neutrophils (Neu#, ×10^9^/L), lymphocytes (Lym#, × 10^9^/L), neutrophil-to-lymphocyte ratio (NLR). An automatic hematological analyzer (BC-6000 Plus, Mindray, Shenzhen, China) was used for these analyses. The tumor volume was calculated by the following formula:

tumor volume = length × (width)^2^/2 (6)

### 3.10. Statistical Analysis

All data are given as mean ± SD. Data were processed using SPSS software (version 11.0; SPSS, Chicago, IL, USA), and one-way ANOVA was performed for statistical multiple groups comparison. *p*-value < 0.05 indicated statistical significance.

## 4. Conclusions

The present study reports a BC-targeted ATG-loaded nanoliposome. SA-ODA was successfully synthesized and employed as a functional phospholipid to prepare SA-decorated liposome (SA-Lip) encapsulating ATG. ATG@Lip, and ATG@SA-Lip with similar size, EE, and DL by the film dispersion method, and its sustained release behavior was thoroughly investigated and compared with ATG@Sol. The formulation and process parameters were systematically studied and optimized. Parameters for these drug-loaded liposomes, including drug loading, stability, and drug release, were evaluated.

The cytotoxicity and antitumor effect of the formulations were also evaluated. ATG@Lip and ATG@SA-Lip inhibited the cell viability of MCF 7 and 4T1 in a dose-dependent manner. The more potent cytotoxicity of ATG@SA-Lip might be due to more cellular uptake by specific receptor-mediated endocytosis.ATG@SA-Lip suppressed tumor growth similar to the control (taxol) treated group. These findings indicate that this sialic acid–octadecylamine conjugate holds promise for preparing liposomal ATG with enhanced safety and anticancer efficiency.

## 5. Patents

There are patents resulting from the work reported in this manuscript.

## Figures and Tables

**Table 1:** Stability of resultant liposomes in plasma (means (± SD), *n* = 3).

Incubation Time (h)	ATG@Lip		ATG@SA-Lip	
	Size (nm)	PDI	Size (nm)	PDI
0	97.4 (±2.1)	0.258	101.1 (±3.7)	0.253
3	89.8 (±3.0)	0.269	99.4 (±4.9)	0.181
6	86.6 (±4.3)	0.286	88.5 (±2.7)	0.269
12	92.0 (±4.1)	0.250	92.0 (±4.1)	0.250
24	88.7 (±5.4)	0.268	86.4 (±3.0)	0.271
48	88.7 (±0.3)	0.247	92.6 (±4.2)	0.257

**Table 2:** Serum biochemistry in mice after different treatment for 14 days (means (± SD), *n* = 3).

Title 1	Normal	NC	Positive	ATG Solution	ATG@Lip	ATG@SA-Lip
HGB	150.0 (±3.0)	143.0 (±5.6)	140.7 (±6.0) *	148.7 (±3.8)	150.7 (±4.6)	149.7 (±2.1)
RBC	>7.90	>7.90	>7.90	>7.90	>7.90	>7.90
WBC	7.42 (±0.7) ^△^	145.8 (±28.9) *	62.1 (±3.9) *^△^	61.0 (±17.1) ^△^	114.9 (±13.0) *	81.1 (±17.9) *^△^
PLT	885.3 (±9.7)	716.3 (±47.4)	879.0 (±70.7) ^△^	793.5 (±58.7)	878.5 (±102.5)	815.3 (±43.8)
Mono#	0.08 (±0.03) ^△^	18.5 (±7.2) *	8.54 (±9.55)	0.47 (±0.30) ^△^	9.26 (±8.28)	9.05 (±2.71)
Bas#	0.03 (±0.04)	0.07 (±0.05)	0.05 (±0.04)	0.01 (±0.01) ^△^	0.08 (±0.03)	0.05 (±0.02)
Eos#	0.11 (±0.03) ^△^	3.14 (±1.00) *	0.49 (±0.23) ^△^	0.87 (±0.51) ^△^	2.18 (±0.64) *	1.68 (±0.90) *^△^
Neu#	1.21 (±0.14) ^△^	109.1 (±18.5) *	48.6 (±36.4) *^△^	38.2 (±24.6) ^△^	90.1 (±15.6) *	58.4 (±11.8) *^△^
Lym#	5.99 (±0.66) ^△^	15.0 (±2.6) *	7.04 (±2.05) ^△^	7.26 (±2.76) ^△^	13.3 (±1.1) *	11.9 (±2.57) *
NLR	0.20 (±0.02) ^△^	7.30 (±0.26) *	6.24 (±3.46) *^△^	4.89 (±2.10) *	6.79 (±1.16) *	4.94 (±0.21) *^△^

HGB, hemoglobin (g/L); RBC, red blood cell (10^12^/L); WBC, white blood cell (10^9^/L); PLT, platelet (10^9^/L), Mono#, monocyte count (10^9^/L), Bas#, basocyte count (10^9^/L); Eos#, eosinophil count (10^9^/L); Neu#, neutrophil count (10^9^/L); Lym#, lymphocyte count (10^9^/L); NLR, neutrophil-to-lymphocyte ratio. Automatic hematological analyzer (BC-6000 Plus, China) was used. * *p* < 0.05 compared with normal; ^△^ *p* < 0.05 compared with NC.

## Data Availability

Data are contained within the article and Supplementary Materials.

## Supplementary Materials

The following supporting information can be downloaded at https://www.mdpi.com/article/10.3390/molecules29010278/s1: Table S1. H’j and W’j values for encapsulation efficiency, drug loading, and particle size after process optimization; Table S2. List of dependent and independent variables in Box–Behnken design; Table S3. Design and results of Box–Behnken design; Table S4. Variance analysis of score in BBD; Table S5. Characterization of optimized liposomes (mean ± SD, *n* = 3); Table S6. Fitting results of release behavior of different formulations in a medium at pH 7.4 and 5.2 (*n* = 3); Table S7. Tumor inhibition rate in mice after different treatment for 14 days.
