# Supplementary material for: Targeted Delivery of Arctigenin Using Sialic Acid Conjugate-Modified Liposomes for the Treatment of Breast Cancer
Source: Molecules. 2024 Jan 4;29(1):278. doi: 10.3390/molecules29010278 (PMC10781120; doi:10.3390/molecules29010278)
Supplement: Supplementary file 1 [file molecules-29-00278-s001.zip › molecules-2758611-supplementary.pdf]

Table S1. H'_j and W'_j of encapsulation efficiency, drug loading, and particle size after process optimization

Indicators	H'_j	W'_j
Encapsulation rate (ER,%)	0.914	0.352
drug loading (DL,%)	0.912	0.362
particle size (Size, nm)	0.930	0.287

The entropy weight method is an objective empowerment method that can be used to calculate the weight of each evaluation index in a complex system. For an evaluation index, the smaller the information entropy (Hj), the larger the weight coefficient (Wj), and the greater the role in the comprehensive evaluation; otherwise, the smaller the index plays in the comprehensive evaluation. The calculation steps are performed as follows:

(1) Undimensionless treatment of the original matrix

Due to the incommensurability among multiple evaluation indicators, the raw data needs to be dimensionless. Assuming a total of a formulation prescription and b evaluation index, the original matrix X= $(x_{ij})_{ab}$, where x_{ij} represents the value of the jth evaluation index of the ith formulation prescription.

For the positive indicator, the formula is:

$$y_{ij} = \frac{x_{ij} - (x_{ij})_{min}}{(x_{ij})_{max} - (x_{ij})_{min}}$$

For the negative indicator, the formula is:

$$y_{ij} = \frac{(x_{ij})_{max} - x_{ij}}{(x_{ij})_{max} - (x_{ij})_{min}}$$

Where i represents the sample value (i=1,2,3,..., a), and j represents the index value (j=1,2,3,..., b). The undimensionalized matrix is Y= $(y_{ij})_{ab}$. They are the positive indicator formula for the EE and drug load, meaning the better the sample prescription; while for the negative indicator, the smaller the y_{ij}, the better the sample prescription.

(2) Calculate the specific weight of P_{ij}

Convert the dimensionless matrix into a probability matrix (P_{ij}):

$$P_{ij} = \frac{y_{ij}}{\sum_{i=1}^{n} y_{ij}}$$

(3) Calculate the entropy H_j

H_j is the entropy of the j-th evaluation index:

$$H_j = -\frac{1}{\ln n}\sum_{i=1}^{n} P_{ij} \ln P_{ij}$$

(4) Calculate the weight W_j

W_j represents the weight of the j th evaluation index:

$$W_j = \frac{g_j}{\sum_{i=1}^{n} g_j}$$

$$(g_j = 1 - H_j)$$

Calculate the composite score (v_j):

$$v_j = \sum_{i=1}^{m} W_j\, y_{ij}$$

Table S2. List of dependent and independent variables in the Box-Behnken Design

Independent Variables		Levels		
		Low (-1)	Intermediate (0)	High (1)
X_1	drug-to-lipid mass ratio (w/w)	1:25	1:20	1:15
X_2	volume of aqueous phase (mL)	5	7.5	10
X_3	ultrasound time (min)	2	6	10
Dependent variables			Goal	
Y_1	ER, %		higher	
Y_2	DL, %		higher	
Y_3	Size, nm		lower	

Table S3. Design and Results of the Box-Behnken Design

Num	Independent Variables			Responses			Score
	X_1	X_2	X_3	Y_1	Y_2	Y_3	
1	-1	0	1	81.8	3.29	101	23.0
2	-1	1	0	85.5	3.29	109	29.0
3	1	-1	0	87.2	5.21	105	73.3
4	1	0	-1	86.5	5.18	134	52.3
5	0	0	0	90.7	4.38	110	65.2
6	-1	0	-1	89.8	3.54	138	29.3
7	-1	-1	0	90.4	3.57	97.4	57.1
8	0	-1	1	89.5	4.24	91.6	70.4
9	0	0	0	87.7	4.25	104	57.1
10	0	0	0	88.9	4.20	105	59.3
11	0	1	-1	83.7	4.00	137	19.8
12	0	1	1	82.4	3.77	101	33.7
13	0	0	0	88.7	4.09	105	56.6
14	0	0	0	89.1	4.34	107	61.6
15	1	0	1	82.5	4.98	95.6	60.2
16	0	-1	-1	93.1	4.49	127	64.5
17	1	1	0	83.5	4.70	114	46.7

Table S4. Variance analysis of Score in BBD

Source of Variation	Sum of Squares	Degrees of Freedom	Mean Square Value	*F*-Value	*P*-Value	
Model	4394	9	488.2	12.9	**0.0014**	Very significant
X_1	1109	1	1109.0	29.2	**0.0010**	
X_2	2318	1	2317.5	61.0	**0.0001**	
X_3	57.3	1	57.3	1.51	0.2588	
X_1X_2	0.516	1	0.516	0.0136	0.9105	
X_1X_3	50.0	1	50.0	1.32	0.2890	
X_2X_3	15.6	1	15.6	0.411	0.5421	
X_1^2	216	1	216.04	5.69	0.0485	
X_2^2	7.01	1	7.01	0.185	0.6804	
X_3^2	566	1	565.6	14.9	**0.0062**	
Residual	265	7	38.0			
Lack of fit	216	3	72.0	5.78	0.0617	not significant
Pure Error	49.8	4	12.5			
Cor total	4660	16				

Table S5. Characterization of optimized liposomes (mean±SD, n=3)

	pH	Size(nm)	PDI	ζ(mV)	EE (%)	DL (%)
ARG@Lip	6.35 ± 0.04	97.4 ± 2.1	0.258 ± 0.003	20.6 ± 2.0	90.3 ± 0.5	4.80 ± 0.04
ARG@SA-Lip	6.49 ± 0.03	101.1 ± 3.7	0.254 ± 0.004	15.6 ± 1.6	91.4 ± 0.4	4.54 ± 0.02

Table S6. Fitting results of release behaviors of different formulations in pH 7.4 and pH 5.2 medium (n = 3)

Formulations	Release model	pH	Fitting equation	R^2
Arg@Lip	Zero-order	7.4	$Q = 2.74t+40.9$	0.558
		5.2	$Q = 2.77\ t + 39.2$	0.588
	First-order	7.4	$Q =86.5(1-e^{-0.3599\ t})$	**0.988**
		5.2	$Q = 86.3(1-e^{-0.3275\ t})$	0.985
	Higuchi	7.4	$Q =17.9\ t^{1/2}+18.7173$	0.801
		5.2	$Q = 17.9\ t^{1/2}+17.1$	0.826
	Ritger - peppas	7.4	$Q = 36.7t^{0.322}$	0.874
		5.2	$Q = 35.1\ t^{0.331}$	0.890
Arg@SA-Lip	Zero-order	7.4	$Q = 3.19\ t+30.2$	0.551
		5.2	$Q = 2.92\ t + 39.7$	0.592
	First-order	7.4	$Q = 87.6\ (1 - e^{-0.246\ t})$	**0.961**
		5.2	$Q = 90.6\ (1-e^{-0.3024\ t})$	0.982
	Higuchi	7.4	$Q = 20.6\ t^{1/2} +4.62$	0.784
		5.2	$Q = 18.9\ t^{1/2} + 16.5$	0.828
	Ritger - peppas	7.4	$Q = 27.7t^{0.407}$	0.814
		5.2	$Q = 35.6\ t^{0.340}$	0.887

Table S7. Tumor inhibition rate in mice after different treatment for 14 days

	In volume (%)	In weight (%)
Positive	65.6 ± 11.9	61.7 ± 10.3
Arg@solution	34.5 ± 8.3**	26.9 ± 8.9***
Arg@Lip	40.8 ± 14.4**	42.1 ± 10.5**
Arg@SA-Lip	51.9 ± 11.7	52.0 ± 7.8

*P<0.05, **P<0.01, ***P<0.001, compared with positive.
